# A new fluorescent and colorimetric chemosensor for Al^3+^ and F^−^/CN^−^ based on a julolidine unit and its bioimaging in living cells†

**DOI:** 10.1039/c8ra05439h

**Published:** 2018-09-04

**Authors:** Fangfang Liu, Congbin Fan, Yayi Tu, Shouzhi Pu

**Affiliations:** Jiangxi Key Laboratory of Organic Chemistry, Jiangxi Science and Technology Normal University Nanchang 330013 PR China congbinfan@163.com pushouzhi@tsinghua.org.cn +86 791 83805212 +86 791 83831996 +86 791 83805212 +86 791 83831996

## Abstract

A novel multifunctional chemosensor HL bearing a julolidine unit and a Schiff base unit has been synthesized. As a fluorescent sensor, HL exhibited excellent selectivity and high sensitivity to Al^3+^ and F^−^/CN^−^ with a low detection limit in acetonitrile. Moreover, HL also showed good colorimetric selectivity to F^−^/CN^−^; a solution color change from colorless to light yellow in acetonitrile was observed by the ‘naked-eye’. The properties of HL with Al^3+^ and F^−^/CN^−^ were studied by UV-vis absorption spectroscopy, fluorescence spectroscopy, high-resolution mass spectrometry and ^1^H NMR titration. Furthermore, the cell imaging experimental results indicated that the chemosensor HL could be applied for the detection of Al^3+^ in living cells.

## Abstract 2

A novel multifunctional chemosensor HL bearing a julolidine unit and a Schiff base unit has been synthesized.

## Graphical abstract

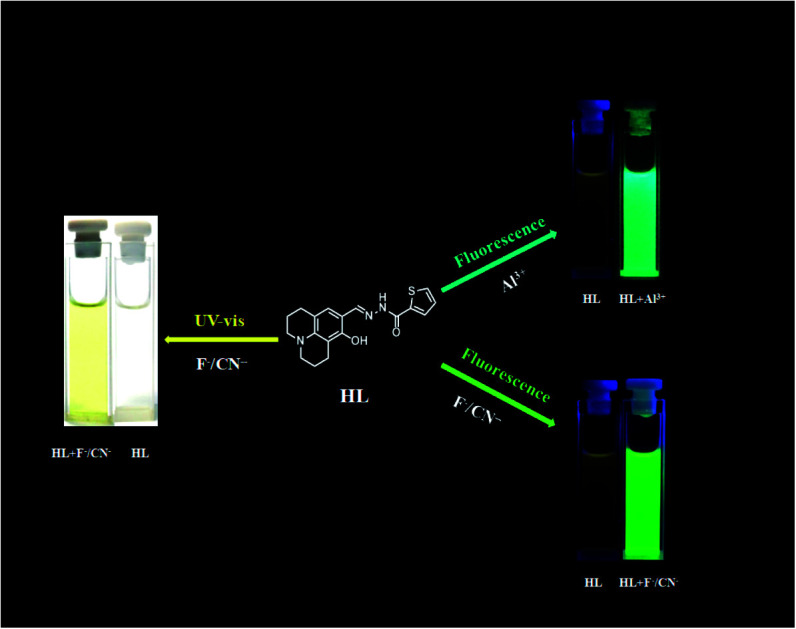

## 1. Introduction

Aluminium is the most prevalent metallic element and the third most abundant element (after oxygen and silicon) in the Earth's crust.^1^ It is widely used in daily life such as in medicines, food additives, and electrical equipment.^2,3^ Besides, it also plays an important role in different industrial fields including water purification, dye and textile production.^4^ In addition, the increase in the concentration of the soluble form (Al^3+^) in water is harmful to aquatic animals and plants because Al^3+^ can acidize water.^5^ World Health Organization (WHO) studies show that the safe daily intake of aluminium is about 3–10 mg.^6^ Excess aluminium homeostasis can cause Alzheimer's disease, Parkinson's disease, dementia, osteoporosis and cancer.^7,8^ For these reasons, it is necessary to detect aluminum in the environment.

In addition, the detection of anions has also aroused much attention in recent years because it plays an important role in medical, chemical, biological and environmental processes.^9,10^ Among various anions, the detection of F^−^ and CN^−^ is particularly important, because fluoride is commonly used as an important functional ingredient in pharmaceutical agents and as a necessary material for uranium separation in the nuclear industry.^11^ Fluoride plays a very important role in our body as the right amount of fluoride intake can cure dental problems and osteoporosis. However, excessive intake of fluoride may affect thyroid activity and may also lead to skeletal fluorosis, depression and adverse effects on the immune system.^12^ The WHO has set a maximum allowable fluoride ion limit of 1.5 mg L^−1^ in drinking water.^13^ On one hand, cyanogen salts are widely used in many chemical and industrial processes such as plastic production, resin industry, organic synthesis, metallurgy and gold mining.^14^ Thus, CN^−^ is present around us in different forms. It can be absorbed through the lungs, gastrointestinal track and skin, which can cause vomiting, convulsion, loss of consciousness, and even death.^15^ The maximum amount of CN^−^ in drinking water is only 1.9 μM based on WHO research.^16^ Most importantly, it is also crucial to detect F^−^/CN^−^ in chemical, biological and environmental samples.

In recent years, many studies have focused on detecting metal ions and anions with multifarious methods.^17,18^ Compared with other test methods, colorimetric and fluorescent methods are the most attractive for the detection of these analytes because of the advantages of high sensitivity, fast response, convenience and low cost.^19^ Among the reported colorimetric and fluorescent sensors, Schiff base sensors are the most common candidates because of their simple synthesis; the nitrogen atom in the C=N bonds have lone pair of electrons which can easily bond with metal ions.^20^ There are many Schiff base chemosensors that can detect Al^3+^ including diarylethene,^21^ naphthalimide,^22^ and thiazole^23^ derivative. In addition to the Schiff base sensors, other types of optical sensors for detecting Al^3+^ have also exhibited significant progress. A large number of Al^3+^ fluorescent sensors have been reported such as some derivatives including rhodamine derivatives,^24^ phenanthroline derivatives,^25^ tetraphenyl ethylene (TPE)–COOH^26^ and other rare earth metal complexes,^27^ and the researches of these sensors have made good results; however, the synthesis process of them was complicated, or the sensor cannot distinguish Al^3+^ from Ga^3+^.^28^ Moreover, there are many fluorescent or colorimetric sensors for detecting F^−^/CN^−^ including benzimidazole–naphthalene conjugate molecules,^29^ silylated derivatives,^30^ and 2-phenyl-2*H*-1,2,3-triazole derivatives.^31^ Besides, some Schiff base compounds can detect F^−^/CN^−^.^32,33^ However, sensors that can simultaneously recognize Al^3+^ and F^−^/CN^−^ are still relatively uncommon.^34,35^

**Fig. 1:**
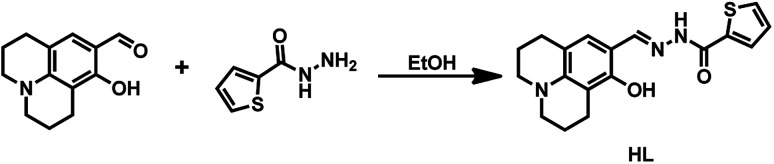
Synthetic route of HL.

Therefore, we designed and synthesized a new Schiff base chemosensor bearing a julolidine unit; the sensor was easy to synthesize and could identify both Al^3+^ and F^−^/CN^−^. Detecting multiple targets with a single receptor is more efficient and less expensive than one-to-one analysis.^36^ The julolidine group is an excellent fluorophore,^37^ and the Schiff base compounds contain a benzhydrazide unit with donor sites such as N atom and O atom for Al^3+^.^38^ Besides, benzhydrazide units contain an amide functional group, and julolidine possesses a phenolic hydroxyl group; the –NH proton and the phenolic hydroxyl group could be linked with F^−^/CN^−^*via* a hydrogen bond to detect F^−^/CN^−^.^39,40^ The synthetic route of sensor HL was shown in Fig. 1. The HL molecule could be easily used as a fluorescent sensor to detect Al^3+^ and F^−^/CN^−^ and as a colorimetric sensor in acetonitrile to recognize F^−^/CN^−^.

## 2. Experimental

### 2.1. General methods

All the solvents were of analytical grade and were not purified before use. Other reagents were of spectroscopic grade. Metal ion solutions were obtained by dissolving the respective nitrates (0.1 M) in distilled water (2.0 mL) except for Hg^2+^ (its counter ion was chloride). All anion solutions were prepared from the dissolution of the corresponding potassium or sodium salts (0.1 M) in distilled water (2.0 mL). NMR spectra were recorded in a Bruker AV400 (400 MHz) spectrometer with DMSO-*d*_6_ as solvent and tetramethylsilane (TMS) as internal standard. UV-vis spectra were measured on an Agilent 8453 UV-vis spectrophotometer. Fluorescence spectra were recorded with a Hitachi F-4600 fluorescence spectrophotometer. Fluorescence quantum yield was measured with an Absolute PL Quantum Yield Spectrometer QY C11347-11. Melting points were obtained on a WRS-1B melting point apparatus. High resolution mass spectra were recorded on an AB SCIEX Triple TOF 4600 instrument. Elemental analysis was performed with a PE CHN 2400 analyzer. Fluorescent cell imaging was obtained on an Olympus FV1000 confocal laser scanning microscope.

### 2.2. Synthesis of HL

8-Hydroxyjulolidine-9-carboxaldehyde (0.11 g, 0.5 mmol) and 2-thiophenecarboxylic hydrazide (0.07 g, 0.5 mmol) were added to ethanol (10 mL) and stirred for 10 min at room temperature. The mixture was heated to reflux for 24 h and then cooled to room temperature; the light yellow precipitate was washed three times with cold ethanol and dried under vacuum to give product HL (0.08 g). Yield 47.06%. Mp 492–493 K. ^1^H NMR (DMSO-*d*_6_, 400 MHz), *δ* (ppm): 11.80 (s, 1H), 11.63 (s, 1H), 8.27 (s, 1H), 7.84–7.83 (d, 2H), 7.21–7.19 (t, 1H), 6.72 (s, 1H), 3.14–3.18 (m, 4H), 2.61–2.49 (m, 4H), 1.84 (s, 4H). ^13^C NMR (DMSO-*d*_6_, 100 MHz): 157.54, 155.27, 151.43, 145.94, 138.63, 132.15, 129.27, 128.99, 128.81, 113.14, 106.96, 106.41, 49.98, 49.52, 27.20, 22.16, 21.34, 20.86. Anal. calcd for C_18_H_19_N_3_O_2_S (%): C, 63.32; H, 5.61; N, 12.31. Found: C, 62.24; H, 5.63; N, 12.34. HRMS: *m*/*z* = 364.1096 [M + Na^+^]^+^ (calcd 364.1095). (Fig. S1–S3†).

### 2.3. Cytotoxicity assay

The HeLa cells were added to a 96-well plate. About 4000 cells per well were placed in a carbon dioxide cell incubator in an atmosphere of 5% CO_2_ and 95% air at 37 °C. Two mg HL was dissolved in DMSO (40 μL) and diluted with DMEM to obtain 5 different concentrations (100, 33.3, 11.1, 3.7, 1.2 μg mL^−1^). Different HL solutions were added to the 96-well plate in a low to high concentration sequence except for that in the control well. Cells were incubated for 24 h to test cytotoxicity. After 24 hours incubation, 20 μL [3-(4,5-dimethylthiazol-2-yl)-2,5-diphenyltetrazolium bromide] (MTT) (5 mg mL^−1^) was added to each well with continuous incubation for another 3.5 h. After MTT was removed, DMSO was added until the compounds were completely dissolved. The absorbance values were measured using a microplate reader. The absorbance of the solvent control cells was considered to be 100%; cell viability and the absorbance of the treated cells were used to calculate the cell viability at different concentrations of HL solution.

### 2.4. Cell culture and imaging

The human cervical HeLa cancer cells were incubated in DMEM (Dulbecco's modified Eagle's medium) supplemented with 10% FBS (fetal bovine serum) and 1% penicillin–streptomycin in the atmosphere of 5% CO_2_ and 95% air at 37 °C. The cells were plated in a cell culture dish overnight. Before the experiments, the cells were washed with phosphate-buffered saline (PBS) buffer. Then, the cells were incubated with HL (20 μM) dissolved in DMEM medium for 30 min at 37 °C. Next, the cells were observed using an Olympus FV1000 confocal laser scanning microscope. Finally, fluorescence images were obtained after incubating the cells with Al^3+^ (50 μM) for another 30 min at 37 °C.

## 3. Results and discussion

### 3.1. Absorption and fluorescence spectral responses of HL toward Al^3+^

**Fig. 2:**
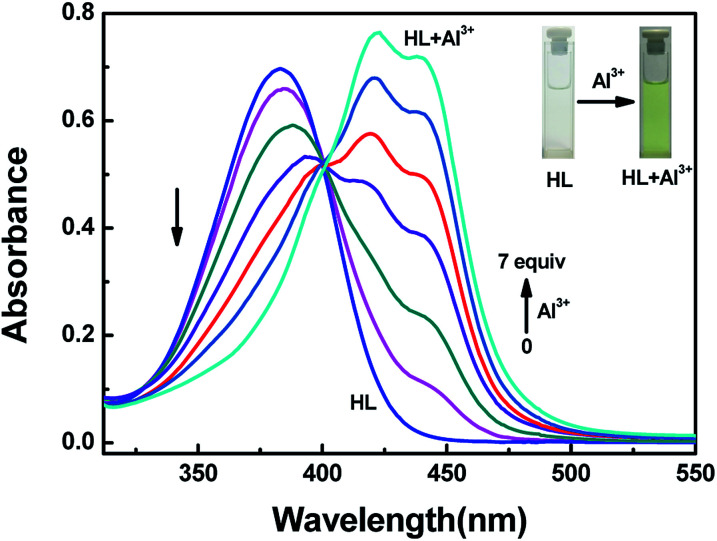
The changes in absorption spectra and color of HL induced by Al^3+^ in acetonitrile (2.0 × 10^−5^ mol L^−1^).

The detection characteristics of the sensor HL for Al^3+^ were preliminarily tested in acetonitrile (2.0 × 10^−5^ mol L^−1^) by a UV-vis spectrometer. Upon addition of Al^3+^ into the solution of HL, a new absorption band appeared, and it was centered at 420 nm and 440 nm. As the concentration of Al^3+^ increased, the absorbance at 420 nm and 440 nm gradually increased, whereas the absorption peak at 384 nm declined slowly. The color of the solution changed from colorless to kelly green (Fig. 2). The curves of absorbance that depended on the equivalents of Al^3+^ increased and then remained stable until the amounts of Al^3+^ reached 7.0 equivalents (Fig. S4†). At the same time, there appeared a clear isosbestic point at 401 nm, indicating the formation of HL–Al^3+^ complex.

**Fig. 3:**
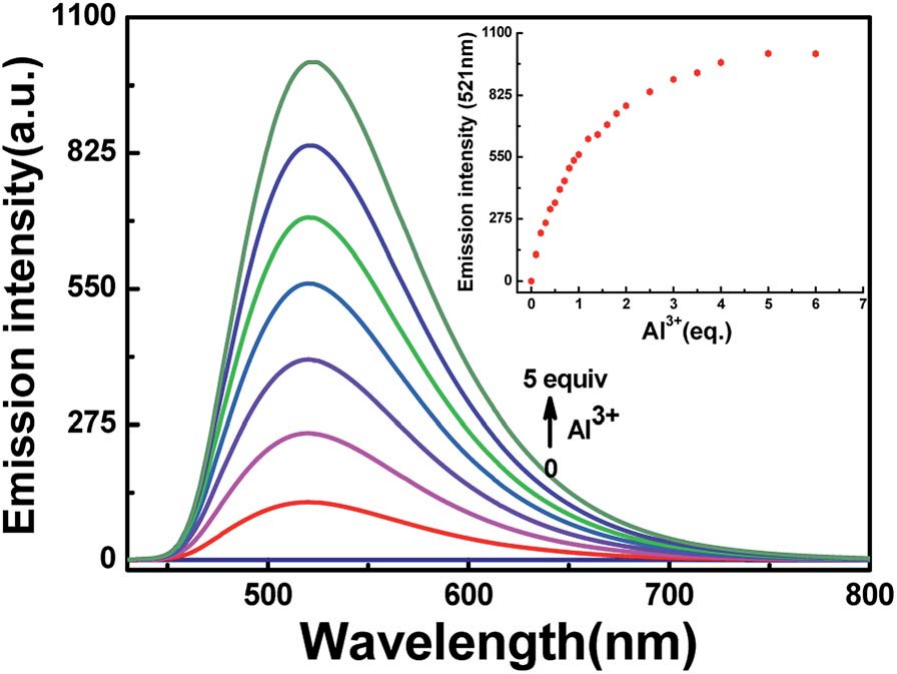
Fluorescence spectral changes of HL induced by Al^3+^ in acetonitrile (2.0 × 10^−5^ mol L^−1^).

To further research the interaction between HL and Al^3+^, fluorescence response experiments were conducted in acetonitrile (2.0 × 10^−5^ mol L^−1^). As shown in Fig. 3, HL exhibited weak fluorescence emission intensity (*Φ* = 0.008) at 420 nm excitation due to C=N isomerization and photoinduced electron transfer (PET) processes.^41,42^ The fluorescence emission intensity of HL at 521 nm enhanced gradually along with the increase in Al^3+^ concentration until the amount of Al^3+^ reached 5.0 equivalents with a high quantum yield (*Φ* = 0.464). Meanwhile, the fluorescence color of HL changed from very faint orange to strong cyan. Compared with the result of HL, the fluorescence intensity of complex HL–Al^3+^ was dramatically enhanced. The phenomenon of fluorescence enhancement was ascribed to the inhibition of C=N isomerization and PET processes.

**Fig. 4:**
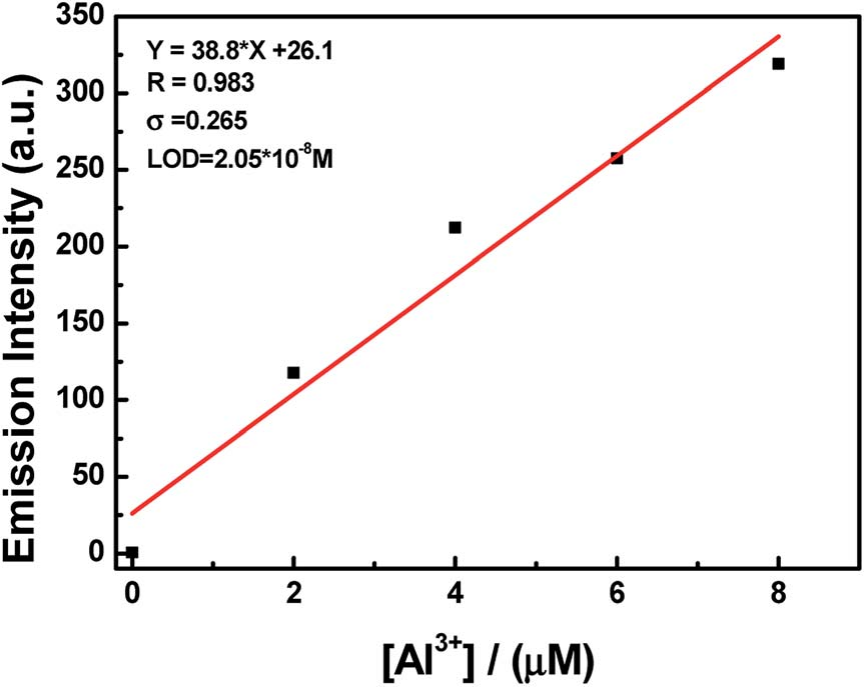
The limit of detection (LOD); LOD is 20.5 nM.

**Scheme 1:**
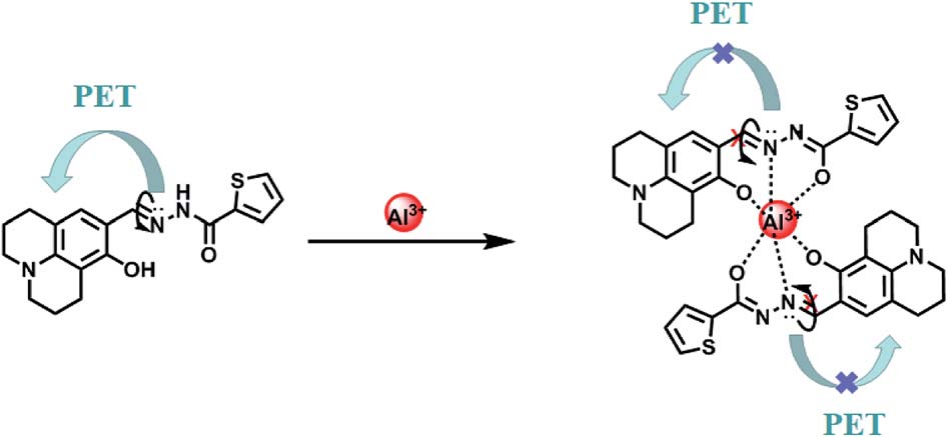
The proposed binding mode of sensor HL with Al^3+^.

To understand the binding characteristics of HL to Al^3+^, the stoichiometry of HL to Al^3+^ was confirmed based on the Job's plot of the fluorescence spectrum (*λ*_em_ = 521 nm). When the molar fraction of (Al^3+^)/[(HL–Al^3+^)] was 0.3, the fluorescence emission intensity value reached its maximum (Fig. S5†); this showed that a 2 : 1 HL–Al^3+^ complex was generated. The association constant (*K*_a_) of HL–Al^3+^ was calculated to be 6.35 × 10^4^ M^−1^ (*R* = 0.996) (Fig. S6†) from the Benesi–Hildebrand equation of the fluorescence titration data.^43^ In addition, the limit of detection (LOD) was measured to be 20.5 nM according to the equation LOD = 3*σ*/*s*, where *σ* is the standard deviation of the blank signal, and *s* is the slope of the linear calibration plot (Fig. 4). Compared with the detection limit of Al^3+^ Schiff base sensors reported in the literatures,^44–49^ the value was relatively low, as shown in Table S1.† These results indicated that HL could be used as a highly sensitive fluorescent sensor to detect Al^3+^. Moreover, high-resolution mass spectrometry analysis of HL in the presence of Al^3+^ was performed; the HRMS peak at *m*/*z* = 705.1917 of [2HL + Al^3+^ − 4H^+^]^−^ (Fig. S7†) confirmed the 2 : 1 stoichiometry between HL and Al^3+^ in acetonitrile. Thus, the proposed sensing mechanism for the detection of Al^3+^ by HL is the chelation of Al^3+^ with the O atom of Ar–OH, the N atom from the –CH=N– group and the O atom of the –C=O moiety, leading to hindering of PET processes. In addition, the isomerization of the C=N bond was suppressed, which stabilized the chelate complexation of HL with Al^3+^ and increased the rigidity of the molecule, thereby causing a chelation-enhanced fluorescence (CHEF) effect.^50–52^ (Scheme 1).

**Fig. 5:**
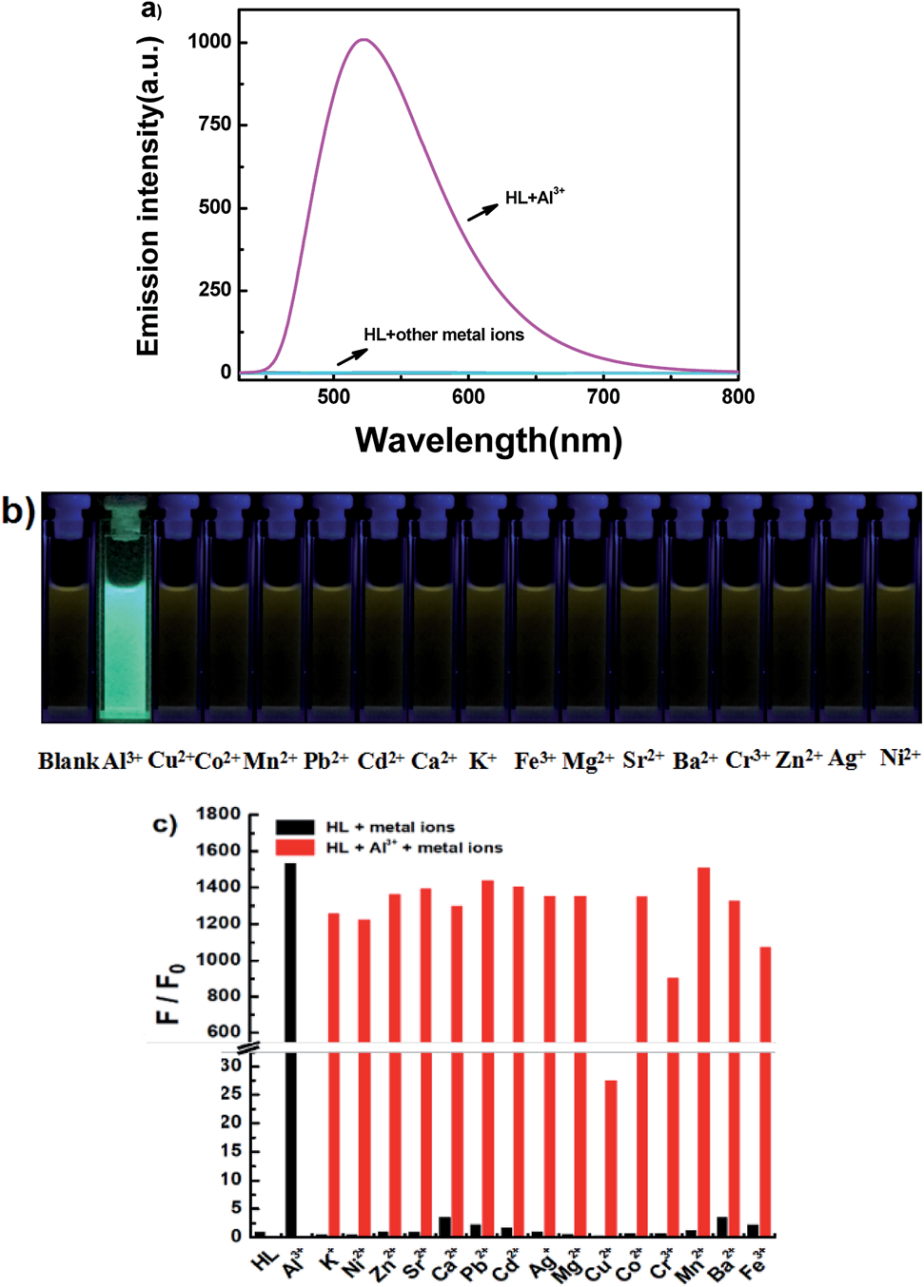
(a) Fluorescence emission spectral changes of HL induced by various metal ions (5.0 equivalents) in acetonitrile (2.0 × 10^−5^ mol L^−1^) by excitation at 420 nm; (b) photos of fluorescence changes in acetonitrile; (c) the fluorescence intensity at 521 nm of HL upon addition of various metal ions (black bars: HL with metal ions; red bars: HL with other metal ions and Al^3+^) in acetonitrile.

The fluorescence spectra of HL in the presence of diverse metal ions (5.0 equiv.) were obtained in acetonitrile (2.0 × 10^−5^ mol L^−1^), and the ions included Cu^2+^, Zn^2+^, Cr^3+^, Al^3+^, Co^2+^, Mn^2+^, Pb^2+^, Cd^2+^, Ca^2+^, K^+^, Fe^3+^, Mg^2+^, Sr^2+^, Ba^2+^, Ag^+^ and Ni^2+^ (Fig. 5). After the addition of equal amounts of metal ions, the fluorescence emission intensity of HL at 521 nm increased sharply only when Al^3+^ was added to the solution at 420 nm excitation (Fig. 5a), and the fluorescence color of the solution changed from faint orange to strong cyan; however, its fluorescence intensity and the color of the solution hardly changed in the presence of other metal ions (Fig. 5b). Subsequently, a competitive experiment was carried out in the presence of the above mentioned metal ions (5.0 equiv.) (Fig. 5c). All these fluorescence emission intensities were similar to that of Al^3+^ except for the result of Cu^2+^, which could be due to the paramagnetic effect on account of spin–orbit coupling. These results showed that HL had excellent selectivity for Al^3+^ in acetonitrile.

### 3.2. Absorption and fluorescence spectral responses of HL to F^−^ and CN^−^

**Fig. 6:**
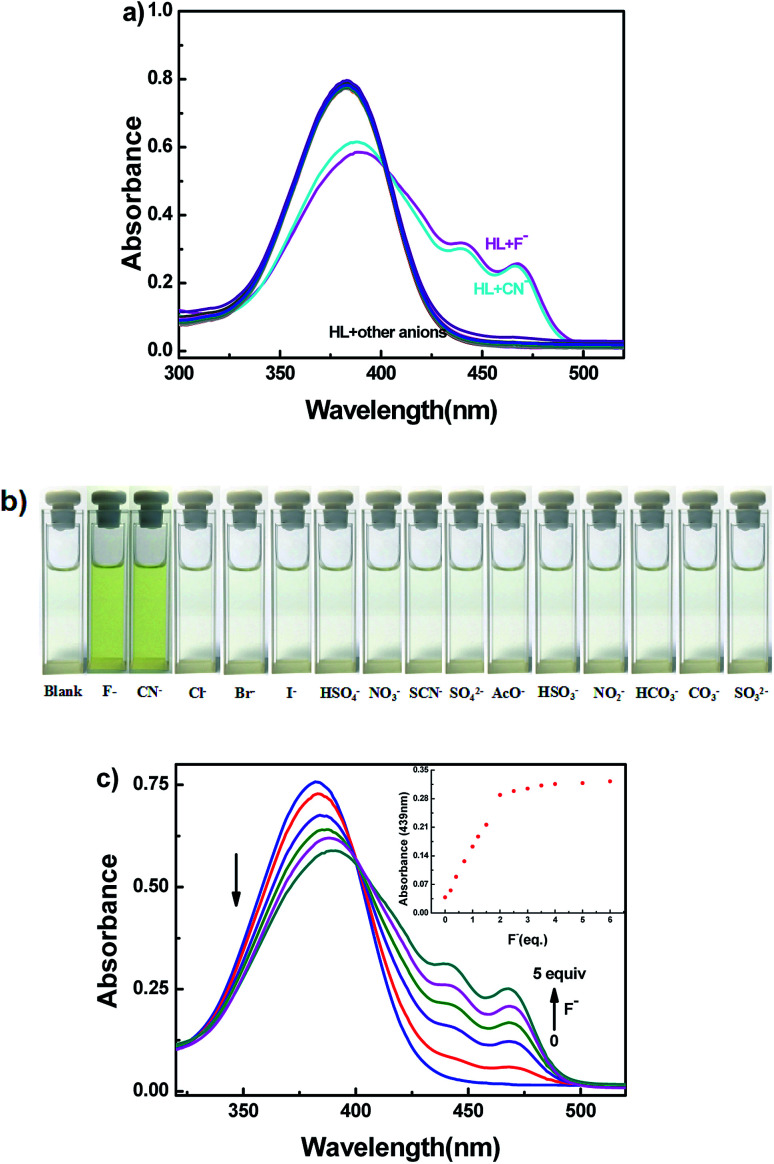
(a) Absorption spectral change of HL induced by various metal ions (5.0 equiv.) in acetonitrile (2.0 × 10^−5^ mol L^−1^); (b) photos of color changes in acetonitrile; (c) absorption spectra and color of HL induced by F^−^ in acetonitrile (2.0 × 10^−5^ mol L^−1^).

The selective experiment of HL to anions was investigated first by UV-vis absorption spectra. Miscellaneous anions including F^−^, CN^−^, Cl^−^, Br^−^, I^−^, HSO_4_^−^, NO_3_^−^, SCN^−^, SO_4_^2−^, AcO^−^, HSO_3_^2−^, NO_2_^−^, HCO_3_^−^, CO_3_^2−^ and SO_3_^2−^ were analyzed individually with the sensor HL in acetonitrile (2.0 × 10^−5^ mol L^−1^) at room temperature (Fig. 6). Upon addition of 5.0 equivalents of other anions to HL, no clear changes in UV-vis absorption spectra were observed; only the addition of the F^−^/CN^−^ solution of HL resulted in the appearance of two new absorption peaks centered at 439 nm and 467 nm (Fig. 6a). At the same time, the color of the HL solution changed from colorless to visible light yellow (Fig. 6b). Other anions caused inappreciable changes of the absorption spectra, and the color of the solution of HL did not change. The absorption spectral change of HL induced by F^−^ in acetonitrile (2.0 × 10^−5^ mol L^−1^) is shown in Fig. 6c. When the amount of F^−^ increased from 0 to 5.0 equivalents, the absorption peaks at 439 nm and 467 nm gradually increased and reached a maximum at 5.0 equivalents of F^−^, whereas the absorption peak at 382 nm decreased when the color of the solution changed to light yellow. Moreover, an isosbestic point was observed at 400 nm, indicating that HL–F^−^ complex was generated. The changes in absorption spectra could be due to intramolecular charge transfer (ICT) mechanism *via* the deprotonation effect. The increased negative charge density of the phenol oxygen atom accelerated ICT from O atom (Ar–OH) to aromatic rings.^53,54^

Similarly, CN^−^ was added to the solution of HL; the absorption spectra presented analogous changes to that observed with the addition of F^−^ (Fig. S8a†). The corresponding sensing mechanism was the same as that for F^−^.^55,56^ The results clearly demonstrated the selective characteristic of the HL sensor for the rapid detection of F^−^ and CN^−^, which could be observed by the naked eye.

**Fig. 7:**
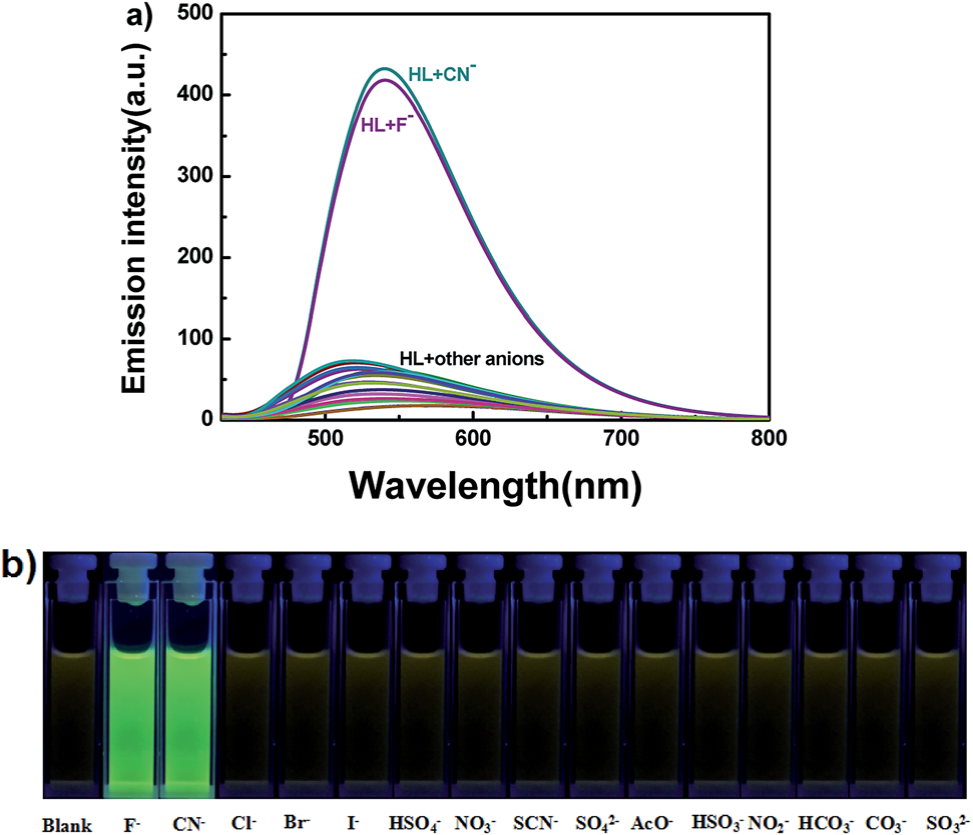
(a) The change in fluorescence spectra of HL induced by various anions (5.0 equiv.) in acetonitrile (2.0 × 10^−5^ mol L^−1^) (excited at 420 nm); (b) photos of color changes in acetonitrile.

**Fig. 8:**
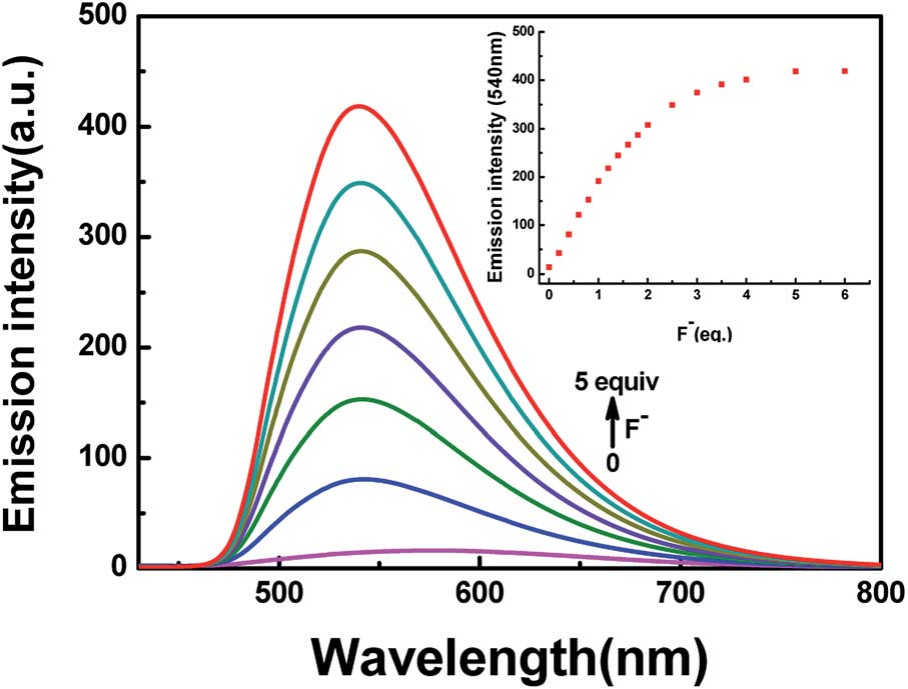
The change in fluorescence emission intensity of HL induced by F^−^ in acetonitrile (2.0 × 10^−5^ mol L^−1^).

In addition to the fluorescence selectivity of HL (2.0 × 10^−5^ mol L^−1^) mentioned above, various anions were also explored in acetonitrile, as depicted in Fig. 7. After addition of F^−^ (5.0 equiv.) to HL, the fluorescence emission intensity enhanced nearly 29-folds at 540 nm (*λ* = 420 nm). On the contrary, there was almost no clear fluorescence signal when other anions were added (Fig. 7a). Meanwhile, the fluorescence color of the solution of HL–F^−^ changed from faint orange to strong green (Fig. 7b). The experiment results indicated that HL could effectively distinguish F^−^ by fluorescence methods. Furthermore, the fluorescent titration interaction between HL and F^−^ was studied in acetonitrile (2.0 × 10^−5^ mol L^−1^), as shown in Fig. 8. On addition of various equivalents of F^−^ (0–5.0 equiv.), the fluorescence emission intensity at 540 nm increased significantly and reached the maximum (*Φ* = 0.021) when the amount of F^−^ reached 5.0 equivalents. Upon continuous addition of F^−^ to the HL solution, the fluorescence spectra hardly changed. The fluorescence spectra of HL–CN^−^ were similar to those of HL–F^−^ (Fig. S8b†). Based on the above results, we inferred that HL can be used as an efficient F^−^/CN^−^ selective fluorescent sensor.

The association constants (*K*_a_) for HL–F^−^ and HL–CN^−^ were obtained as 1.25 × 10^4^ M^−1^ (*R* = 0.995) (Fig. S9†) and 1.48 × 10^4^ M^−1^ (*R* = 0.995) (Fig. S11†), respectively, from the fluorescence titration data. The detection limits of HL for F^−^ and CN^−^ were calculated to be 88.4 nM (Fig. S10†) and 61.0 nM (Fig. S12†), respectively.

**Fig. 9:**
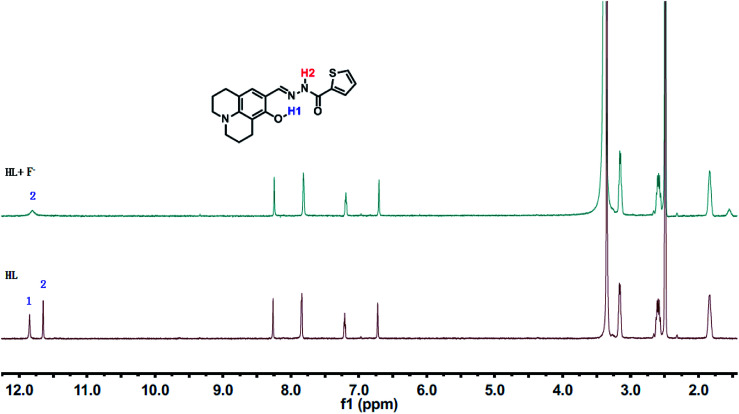
^1^H NMR spectral changes of HL induced by F^−^ in DMSO-*d*_6_.

**Scheme 2:**
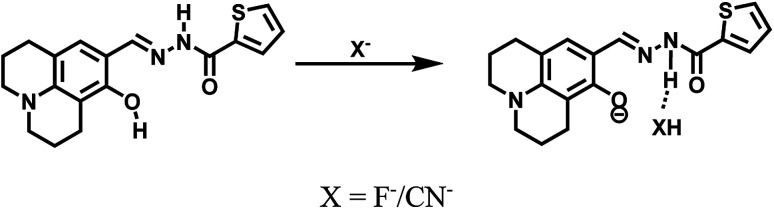
Proposed sensing mechanism of F^−^/CN^−^ by HL.

^1^H NMR titrations were performed in DMSO-*d*_6_ to prove the reaction between HL and F^−^. When excess F^−^ was added to a solution of HL, the signal for –OH (11.80 ppm) completely disappeared. The proton H2 (–N–H) shifted from 11.65 ppm to 11.70 ppm, and the peak intensity decreased (Fig. 9). ^1^H NMR titrations of HL with CN^−^ showed similarities to the results shown above (Fig. S13†). This clearly indicated that the strong hydrogen bond interaction caused deprotonation. The proposed sensing mechanism of F^−^/CN^−^ by HL is shown in Scheme 2.

### 3.3. Cell cytotoxicity and cell imaging

**Fig. 10:**
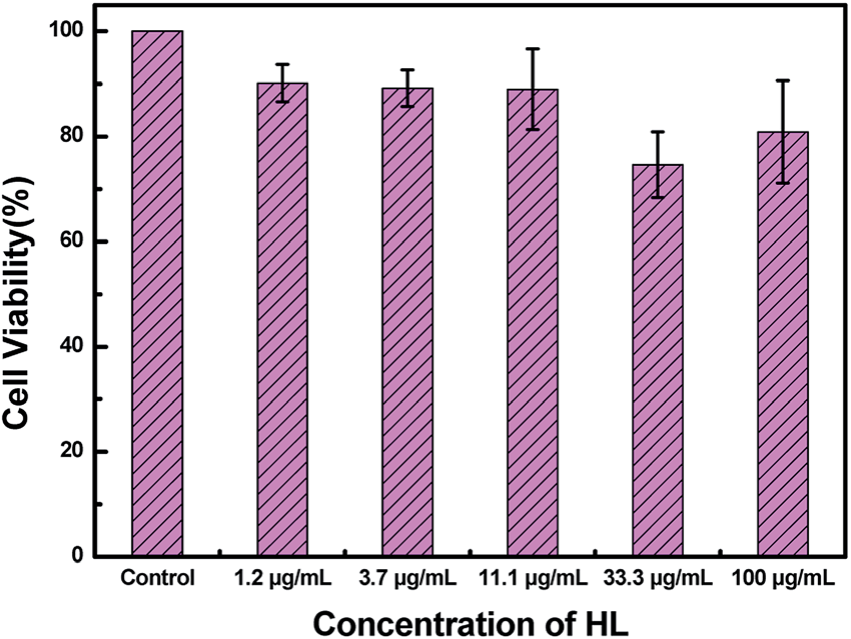
Cell viability of HeLa cells incubated with various concentrations of HL for 24 h.

To measure the cytotoxicity of HL, a cell cytotoxicity experiment by the MTT assay on HeLa cells was carried out. As shown in Fig. 10, the cell viability gradually decreased as the concentration of HL increased. The MTT assay showed that the cell viability was basically over 80%. This result demonstrated that the cytotoxicity of HL was relatively low for fluorescence imaging in living cells.

**Fig. 11:**
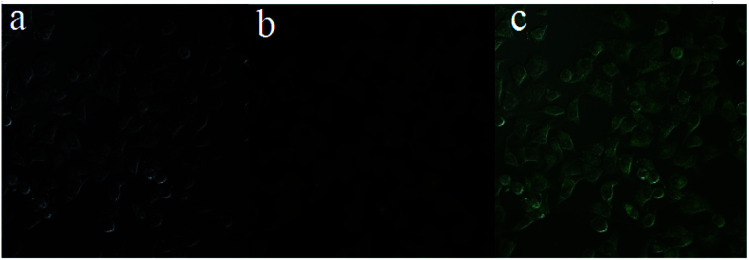
Fluorescence images of HeLa cells. (a) Microscopy image of HeLa cells treated with HL (20 μM); (b) microscopy image of HeLa cells treated with HL (20 μM) in the presence of Al^3+^ (50 μM); (c) merge of (a and b).

Next, to examine the biological applicability of HL to detect Al^3+^ in the biological systems, the sensor HL was applied for fluorescence imaging experiments of intracellular Al^3+^ in living cells. The fluorescence images were taken by a confocal laser microscope. The HeLa cells were incubated with HL (20 μM) for 30 min at 37 °C; almost no fluorescence was observed (Fig. 11a). Nevertheless, the addition of Al^3+^ (50 μM) to the above HeLa cells triggered a striking fluorescence enhancement and resulted in green fluorescence (Fig. 11b). The results demonstrated that the sensor HL could play an indispensable role in intracellular fluorescence imaging of Al^3+^.

## 4. Conclusion

In summary, we have designed and developed a new multiple-ion-responsive Schiff base chemosensor HL, which can simultaneously detect Al^3+^ and F^−^/CN^−^. HL exhibited excellent colorimetric selectivity to F^−^/CN^−^ and highly specific fluorescence responses to Al^3+^ and F^−^/CN^−^. Upon addition of Al^3+^ or F^−^/CN^−^, HL showed clear fluorescence increase with different emission spectra. The results demonstrated that the compound HL could be used as a fluorescent chemosensor to detect Al^3+^ and F^−^/CN^−^. Besides, it could also serve as a colorimetric chemosensor to recognize F^−^/CN^−^*via* a clear color change from colorless to visible light yellow. Furthermore, the relatively low cytotoxicity of HL could be successfully used for cell imaging to identify Al^3+^, indicating its promising application prospects in living cells.

## Conflicts of interest

There are no conflicts to declare.

## Supplementary Material

RA-008-C8RA05439H-s001
