# Supplementary material for: A new fluorescent and colorimetric chemosensor for Al3+ and F−/CN− based on a julolidine unit and its bioimaging in living cells
Source: RSC Adv. 2018 Sep 4;8(54):31113–20. doi: 10.1039/c8ra05439h (PMC9085603; doi:10.1039/c8ra05439h)
Supplement: RA-008-C8RA05439H-s001 [file RA-008-C8RA05439H-s001.pdf]

Electronic Supplementary Material (ESI) for RSC Advances.
This journal is © The Royal Society of Chemistry 2018

Supplementary Information (SI)

A new fluorescent and colorimetric chemosensor for Al^{3+} and F^-/CN^- based on a julolidine unit and its bioimaging in living cells

Fangfang Liu, Congbin Fan,* Yayi Tu and Shouzhi Pu*

Jiangxi Key Laboratory of Organic Chemistry, Jiangxi Science and Technology Normal University, Nanchang 330013, PR China

** Corresponding authors.* Tel./fax: +0791 83805212 (C. Fan), +86 791 83831996 (S. Pu).

E-*mail:* congbinfan@163.com (C. Fan), pushouzhi@tsinghua.org.cn (S. Pu).

Sensor	Solvent	Detection limit	Cell image	Reference
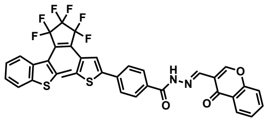	CH_3CN	5.47×10^{-7} M	No	44
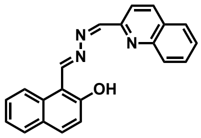	MeOH-HEPES buffer (8/2, v/v)	7.41× 10-6 M	No	45
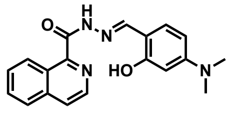	MeOH	8.08×10^{-8} M	Al^{3+}	46
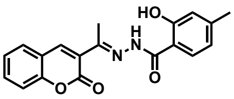	DMF-H_2O (9/1, v/v)	6.7×10^{-6} M	No	47
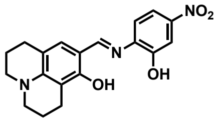	MeOH-H_2O (6/4, v/v)	1.5×10^{-6} M	No	48
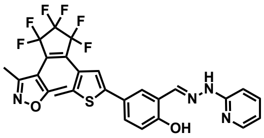	CH_3CN	1.8×10^{-7} M	No	49
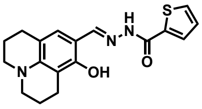	CH_3CN	20.5 nM	Al^{3+}	This work

Table S1. Examples for the detection of Al^{3+} by organic chemosensors.

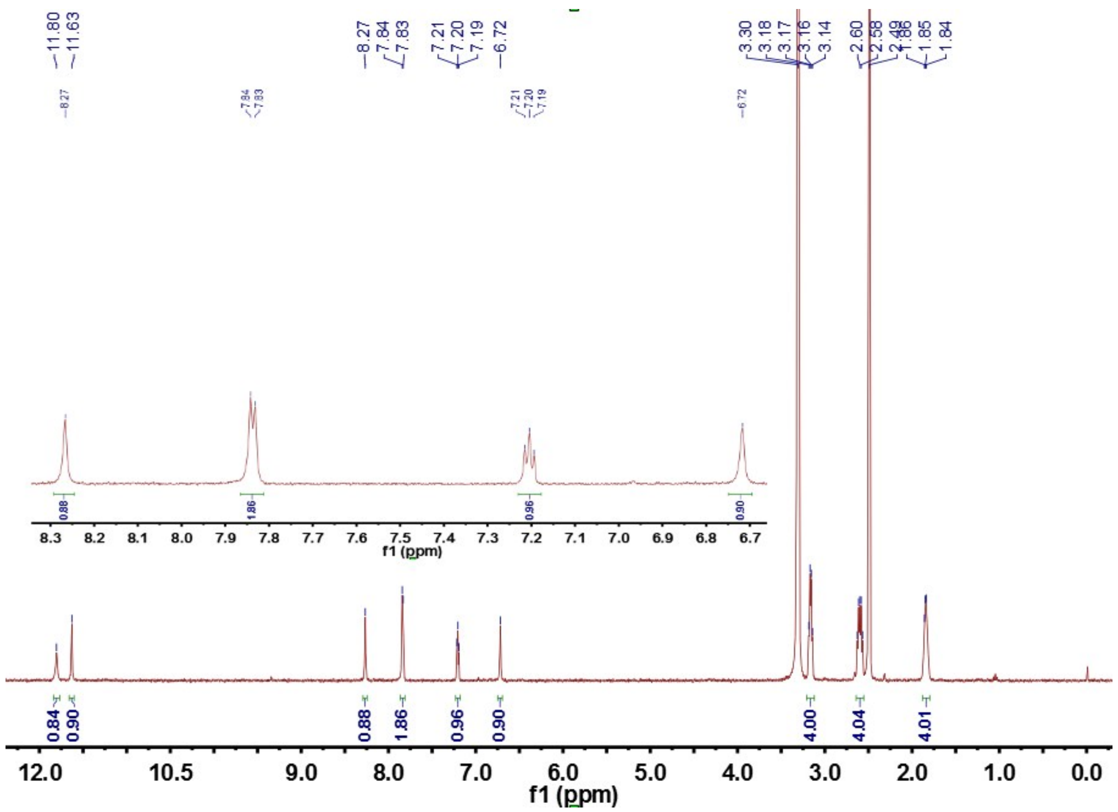

Fig. S1. ^{1}H NMR (DMSO-d_6, 400 MHz) spectrum of **HL**.

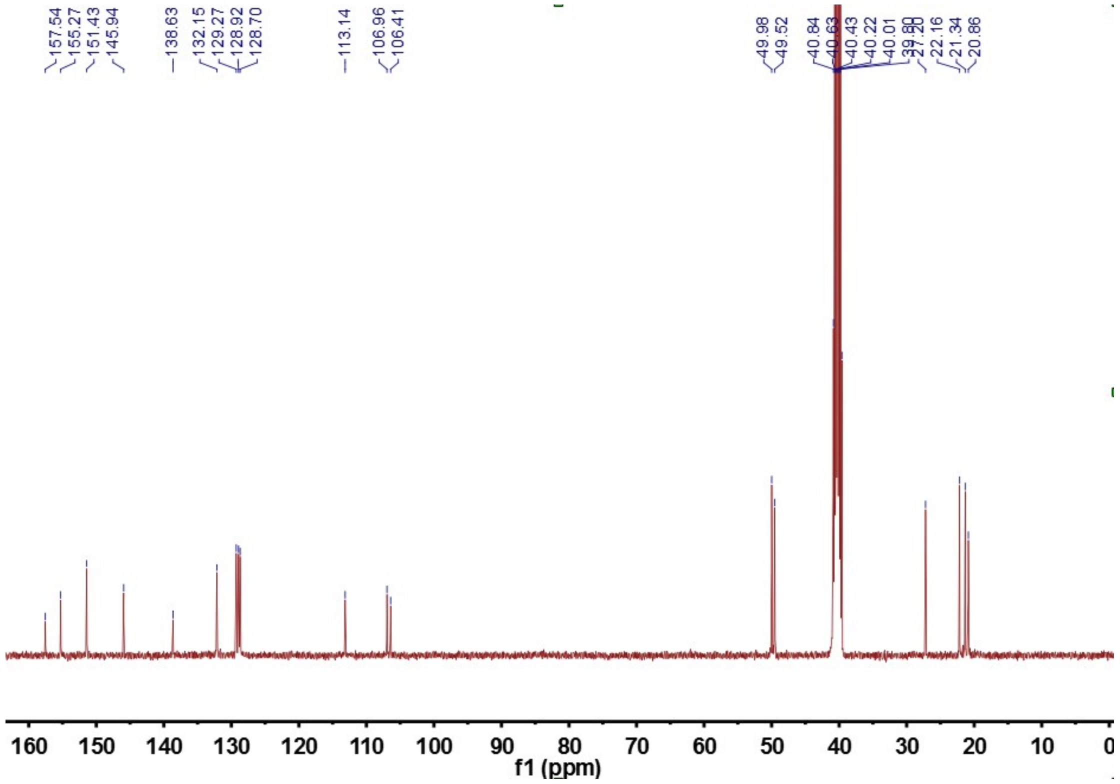

Fig. S2. ^{13}C NMR (DMSO-d_6, 100 MHz) spectrum of **HL**.

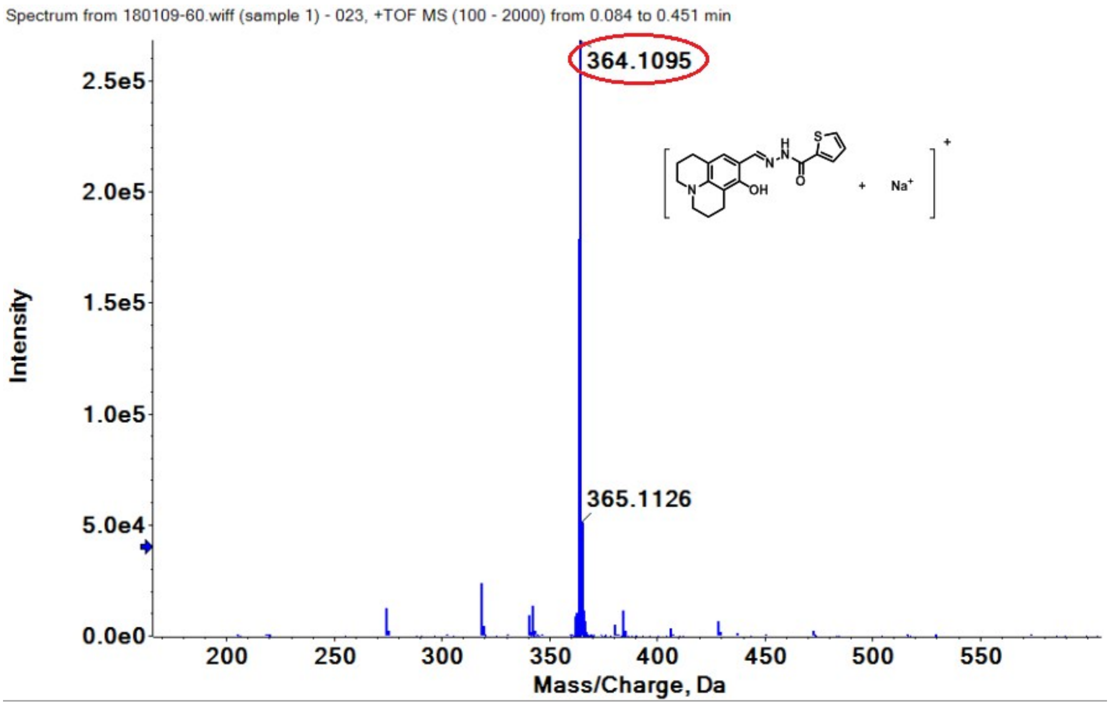

Fig. S3. HRMS of **HL**.

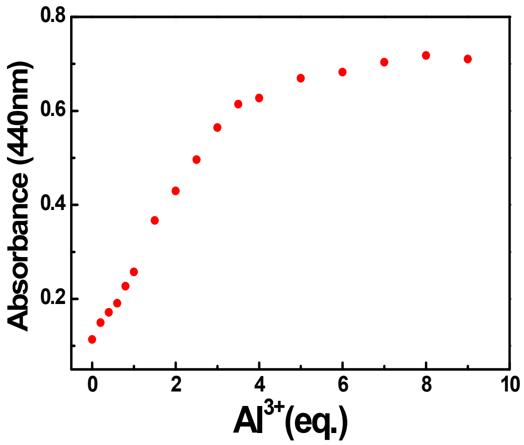

Fig. S4. Absorbance of **HL** at 440 nm in the presence of different equiv. of Al^{3+}.

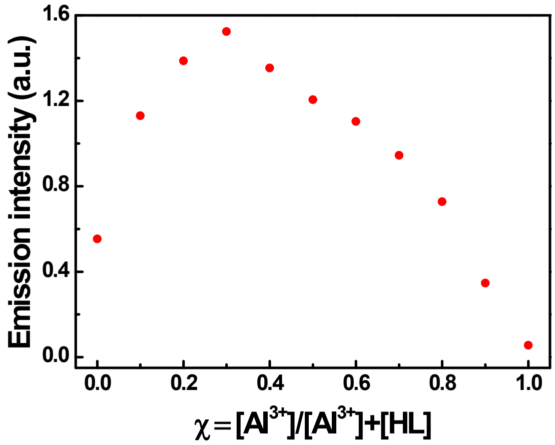

Fig. S5. Job's Plot of fluorescence titration (λ=521nm) of **HL** with Al^{3+}, showing 2:1 stoichiometry.

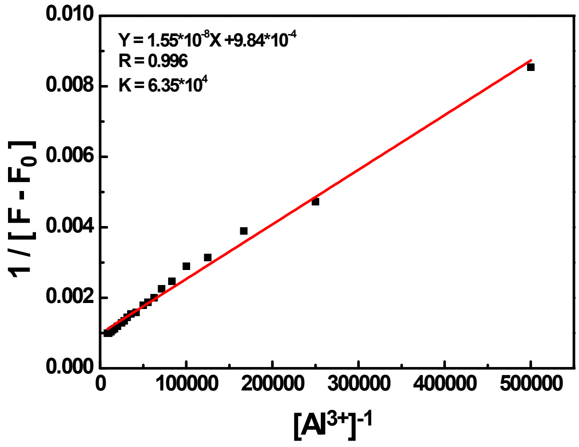

Fig. S6. Hildebrand–Benesi plot based on the 2:1 ratio between **HL** and Al^{3+}, the association constant (K_a) of **HL** with Al^{3+} was calculated to be 6.35×10^4 M^{-1}.

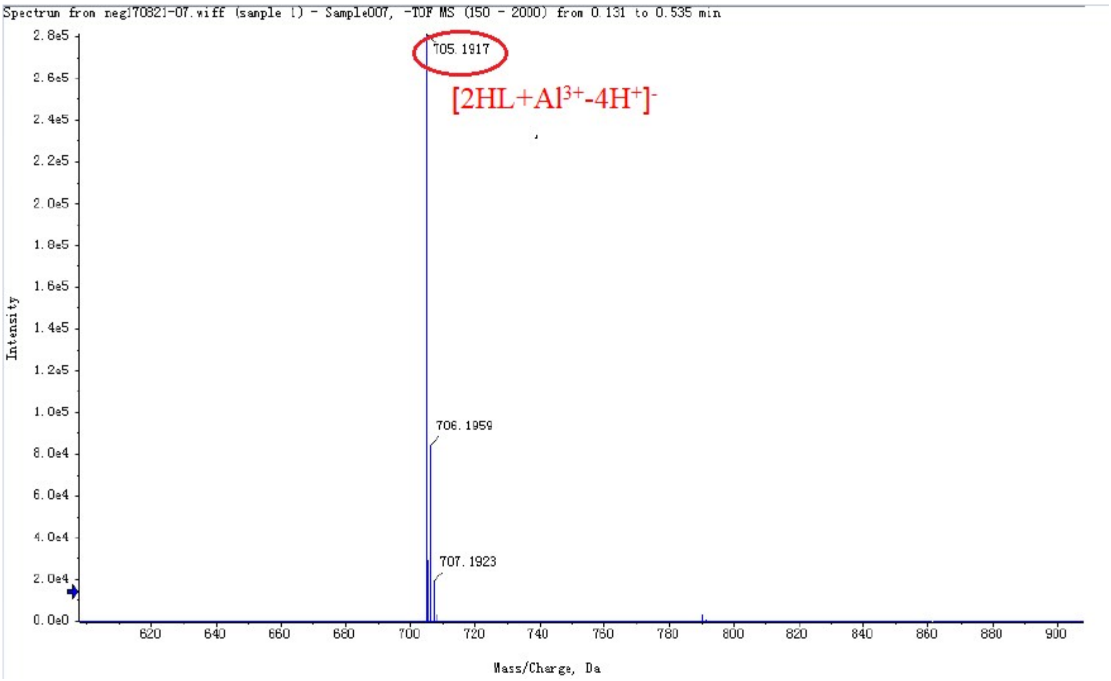

Fig. S7. HRMS of **HL**+Al^{3+}.

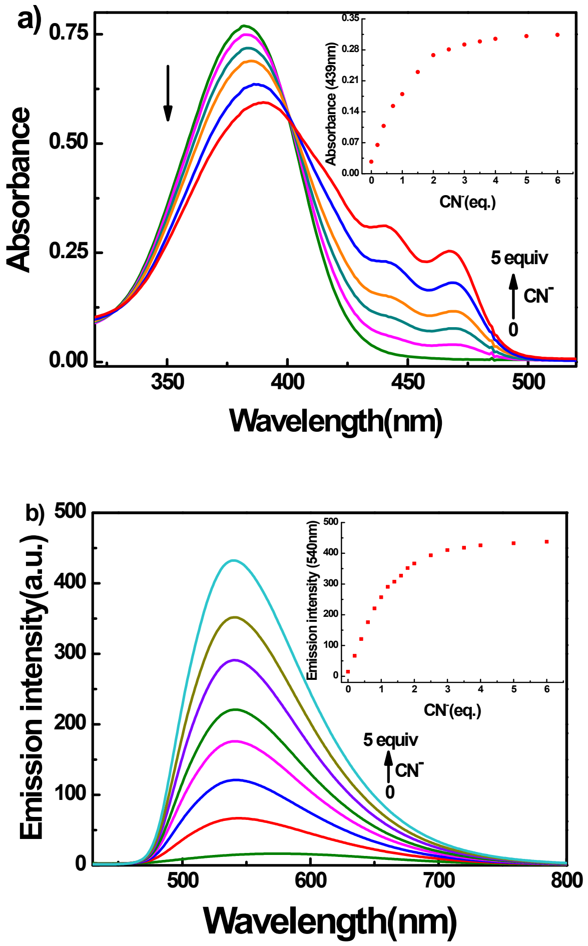

Fig. S8. The change of absorption spectra and color of **HL** induced by CN^{-} in acetonitrile (2.0×10^{-5} mol L^{-1}); b) the change of fluorescence emission intensity of **HL** induced by CN^{-} in acetonitrile (2.0×10^{-5} mol L^{-1}).

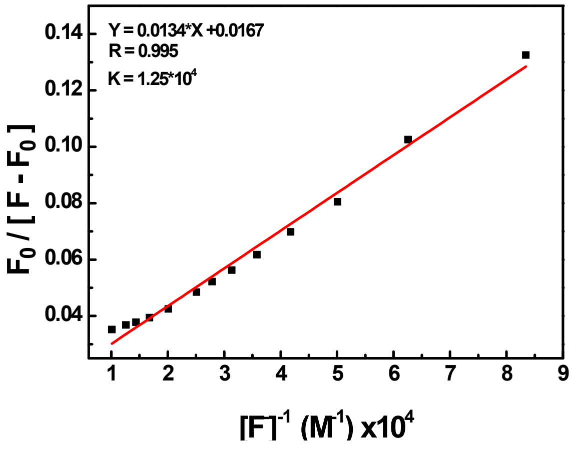

Fig. S9. The association constant (K_a) of **HL** with F^{-} was calculated to be 1.25×10^{4} M^{-1}.

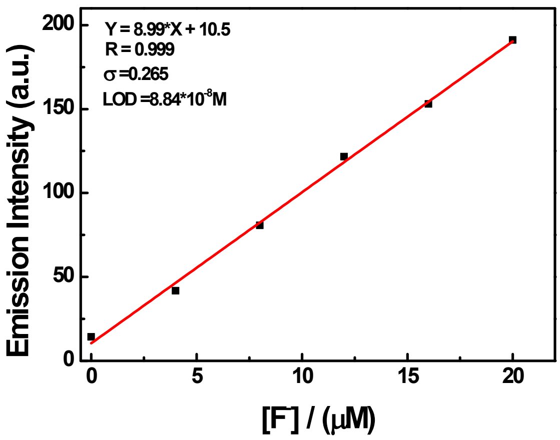

Fig. S10. The limit of detection (LOD) of **HL** toward F^- , LOD is 88.4 nM.

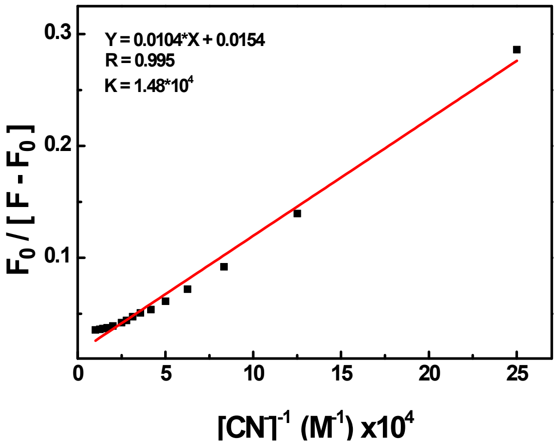

Fig. S11. The association constant (K_a) of **HL** with CN^- was calculated to be 1.48×10^4 M^{-1}.

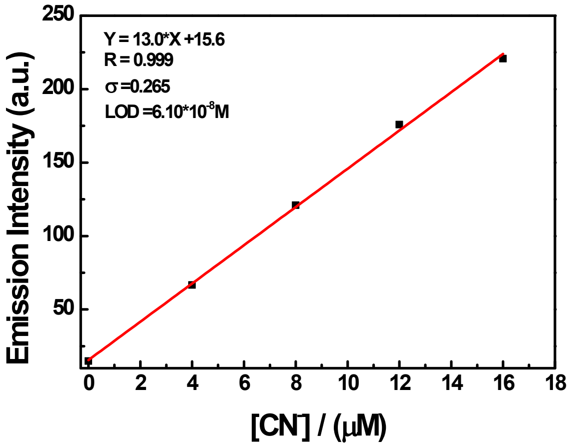

Fig. S12. The limit of detection (LOD) of **HL** toward CN^-, LOD is 61.0 nM.

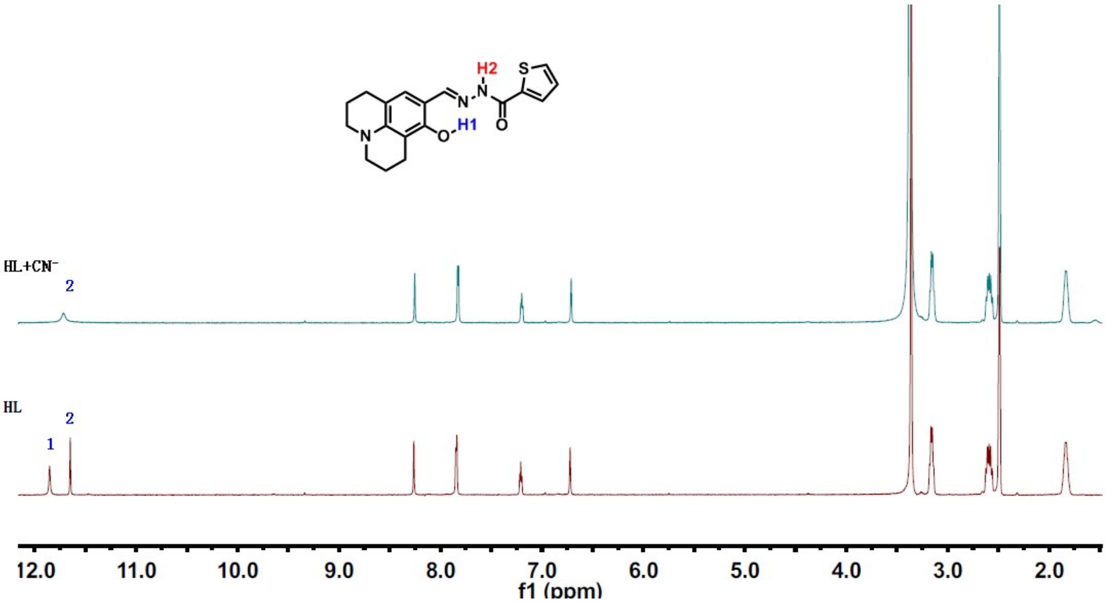

Fig. S13. ^{1}H NMR spectral changes of **HL** induced by CN^- in DMSO-d_6.
